# Wnt/β-Catenin Signaling in Liver Cancers

**DOI:** 10.3390/cancers11070926

**Published:** 2019-07-02

**Authors:** Wenhui Wang, Ron Smits, Haiping Hao, Chaoyong He

**Affiliations:** 1 State Key Laboratory of Natural Medicines, Department of Pharmacology, China Pharmaceutical University, Nanjing 211198, China; 2 Department of Gastroenterology and Hepatology, Erasmus MC-University Medical Center and Postgraduate School Molecular Medicine, Rotterdam 3015 CN, The Netherlands

**Keywords:** liver cancer, precancerous lesion, HCC, CCA, HB, Wnt/β-catenin signaling

## Abstract

Liver cancer is among the leading global healthcare issues associated with high morbidity and mortality. Liver cancer consists of hepatocellular carcinoma (HCC), cholangiocarcinoma (CCA), hepatoblastoma (HB), and several other rare tumors. Progression has been witnessed in understanding the interactions between etiological as well as environmental factors and the host in the development of liver cancers. However, the pathogenesis remains poorly understood, hampering the design of rational strategies aiding in preventing liver cancers. Accumulating evidence demonstrates that aberrant activation of the Wnt/β-catenin signaling pathway plays an important role in the initiation and progression of HCC, CCA, and HB. Targeting Wnt/β-catenin signaling potentiates a novel avenue for liver cancer treatment, which may benefit from the development of numerous small-molecule inhibitors and biologic agents in this field. In this review, we discuss the interaction between various etiological factors and components of Wnt/β-catenin signaling early in the precancerous lesion and the acquired mechanisms to further enhance Wnt/β-catenin signaling to promote robust cancer formation at later stages. Additionally, we shed light on current relevant inhibitors tested in liver cancers and provide future perspectives for preclinical and clinical liver cancer studies.

## 1. Introduction

Liver cancer is among the leading global healthcare issues associated with high morbidity and mortality. Primary liver cancers consist of hepatocellular carcinoma (HCC), cholangiocarcinoma (CCA), and hepatoblastoma (HB). Other rare tumors are fibrolamellar carcinoma, hepatocellular adenoma, focal nodular hyperplasia, fibrosarcoma, angiosarcoma, leiomyosarcoma, and lymphoma.

HCC is the fifth most frequent cancer worldwide and is closely associated with cancer-related deaths [1]. HCC accounts for around 90% of liver cancer patients. The etiological factors for HCC include hepatitis B (HBV), hepatitis C (HCV) viruses, alcohol abuse, obesity-induced non-alcoholic steatohepatitis (NASH), as well as aflatoxin-B1 exposure. Most HCCs start with chronic hepatitis caused by the above etiological factors, which gives rise to fibrosis and progresses to cirrhosis around 10 years later [1].

CCA is the second most common liver cancer following HCC. CCA is a devastating malignancy with a dismal 5-year overall survival rate of less than 10%. Based on the anatomic location, CCAs are classified into intrahepatic (iCCA), perihilar (pCCA), and distal (dCCA) subtypes. iCCA associates better with cirrhosis resulting from HCV than HBV infection. pCCA is closely related to primary sclerosing cholangitis marked by chronic inflammation [2].

HB is the most common pediatric liver malignancy and stems from hepatic progenitor cells that undergo malignant transformation during embryogenesis [3]. Although surgery along with chemotherapy has largely improved prognosis, around one quarter of the patients die of the disease. Compared to the general population, the risk of HB is 750–7500 times higher in children predisposed to familial adenomatous polyposis (FAP) [4].

With the exception of HBs, most liver cancers take decades to facilitate the progression from the precancerous dysplasia of liver cells located in a chronic inflammatory microenvironment towards a malignant phenotype. During this process, the accumulation of aberrant genetic and epigenetic modifications leads to the dysregulation of signaling pathways, which in turn promote the initiation and progression of liver cancers.

Aberrantly activated Wnt/β-catenin signaling plays a critical role in precancerous dysplasia as well as the malignant transformation of liver cells and malignant expansion of cancer cells. Here, we summarize the latest advances in our understanding of Wnt/β-catenin signaling in the course of liver cancer development and highlight the clinical implications of Wnt/β-catenin signaling pathway as a rational therapeutic target.

## 2. Wnt/β-Catenin Signaling

The Wnt/β-catenin signaling pathway is evolutionarily conserved and required in both physiological and pathophysiological processes [5,6,7]. The association of Wnt ligands to corresponding receptors triggers the Wnt/β-catenin signaling pathway. These ligands are generated within the endoplasmic reticulum (ER), which undergoes palmitoylation by the Wnt acyl-transferase porcupine (PORCN). Next, Wntless (WLS) shuttles the palmitoylated ligands from the Golgi to the cell membrane, where they can signal in an autocrine or paracrine manner [8].

Wnt/β-catenin signaling is inactive in the normal tissues of adults, except for some stem cell niches [9]. This is achieved through a balanced regulation through Wnt ligands and the β-catenin destruction complex. Wnt antagonists, including secreted frizzled-related proteins (SFRPs), dickkopfs (DKKs), and the Wnt inhibitory factor (WIF) capturing Wnt ligands, as well as Kallistatin binding to the low-density lipoprotein receptor-related protein 6 (LRP6) inhibit the combination of Wnt ligands and receptors [10,11]. The central component β-catenin is tightly regulated by the destruction complex, which is composed of scaffold proteins AXIN1 and AXIN2, adenomatous polyposis coli (APC), and the kinases GSK3 and CK1α. β-catenin is subject to phosphorylation firstly at Ser45 by CK1α, potentiating the subsequent phosphorylation at Thr41, Ser37, and Ser33 by GSK3. Next, the phosphorylated β-catenin is recognized by the β-transducin repeat containing protein (βTRCP), which mediates its ubiquitylation and subsequent proteolysis [10,12].

**Figure 1:**
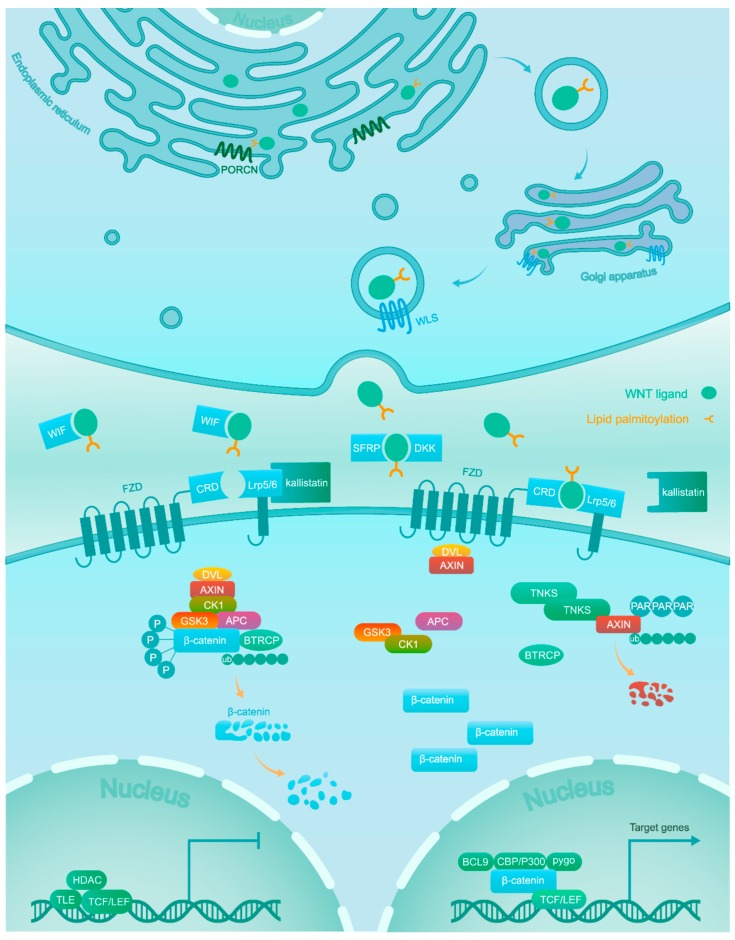
Wnts are lipid-modified by PORCN in the ER and escorted by WLS from the Golgi to the plasma membrane for secretion. In the absence of Wnt ligands due to Wnt antagonists (WIF, DKK, and SFRP) and Kallistatin, β-catenin is phosphorylated by a destruction complex consisting of GSK3, CK1, APC and AXIN. Phosphorylated β-catenin is targeted for proteasomal degradation after ubiquitination. In the nucleus, the TCF/LEF transcription factor activity is repressed by transducin-like enhancer of split (TLE) and histone deacetylase (HDAC). Association of Wnt ligands with their receptors leads to the dissociation of the destruction complex. As a result, β-catenin accumulates in the cytoplasm and translocates into the nucleus, where it promotes the expression of target genes via interaction with TCF/LEF and co-activators such as CBP/300, BCL9, and Pygo.

On the contrary, in the active condition Wnt ligands bind at the cysteine rich domain (CRD) of one of the frizzled (FZD) receptors and one of the LRP5/6 co-receptors. Next, the interaction between Wnt ligands and receptors recruits scaffolding proteins disheveled (DVL) and AXIN to the membrane. The AXIN proteins themselves are also under proteolytic control and are rapidly degraded following poly-ADP-ribosylation (PAR) by tankyrase (TNKS) enzymes [13,14]. The sequestration and degradation of AXIN result in the disassembly of the β-catenin destruction complex [15] and subsequent accumulation of unphosphorylated β-catenin in the cytoplasm, which acquires the ability to escape ubiquitylation and proteolysis. This allows active β-catenin to translocate to the nucleus [16], where it binds to transcription factors of the T-cell factor/lymphoid enhancer-binding factor (TCF/LEF) family with recruitment of co-activators (BCL9, CBP/300, Pygo, and others) to turn on the transcription of downstream target genes [17] (Figure 1).

## 3. Precancerous Lesion

Aberrant activation of Wnt/β-catenin signaling exists at both hepatic precancerous lesions and cancerous foci. Uncovering how the signaling is deregulated in these precancerous lesions will be beneficial to better understand the mechanisms contributing to the progression of liver cancer. The most important event in precancerous lesions is the dysplasia and dysfunction of liver cells infected or affected by the given etiological factors. Thus, it is necessary to elucidate the interaction between these factors and components of Wnt/β-catenin signaling in these cells.

### 3.1. Hepatitis Viruses

#### 3.1.1. HBV

HBV infection increases the risk of developing HCC about 100–200-fold [18]. HBV is a hepadnavirus with double-stranded DNA including four overlapping open reading frames encoding a viral DNA polymerase, two structural proteins (the surface and core antigens), and the regulatory hepatitis B viral X protein (HBx) [19]. Besides maintaining the transcription and replication of HBV, HBx plays an important role in activating Wnt/β-catenin signaling in the infected hepatocytes. HBx regulates multiple components of Wnt/β-catenin signaling at extracellular and intracellular levels. Extracellularly, HBx dramatically diminishes Wnt antagonist SFRP1 and SFRP5 expression due to genetic silencing by recruiting DNA methyltransferase 1 and 3A to gene promoters [20]. Intracellularly, HBx compromises the function of the destruction complex by competitively binding APC [21] or by inhibiting GSK3 activity through activation of Src kinase [22] as well as induction of cell-cycle-related kinase-mediated androgen receptor signaling [23].

The hepatitis B surface antigen (HBsAg) regulates the expression of LEF-1, a key transcription factor of β-catenin in the nucleus [24,25,26]. A marked increase of LEF-1 was observed in HBsAg-expressing HCC cell lines and confirmed by interference experiments with small interfering RNA [25]. Furthermore, the same group validated that HBsAg increased the level of LEF-1 along with c-Myc and cyclin D1 (β-catenin downstream genes), more pronounced in peritumor tissues compared to liver tumors in HBV-associated HCC patients [26].

The integration of viral DNA into the host genome is a special event during HBV infection that occurs mostly near particular sites, e.g., the long interspersed nuclear elements (*LINE*s). The HBV genome inserted into a *LINE1* element produces an oncogenic HBV-*LINE1* chimeric transcript, inducing nuclear localization of β-catenin and triggering target gene expression [18,27]. Nevertheless, the fusion transcripts were not detectable in other cohorts [28,29], and therefore the observation needs further investigation in more cohorts from different regions.

Despite a major risk factor for CCA, HBV function on Wnt/β-catenin signaling in infected cholangiocytes remains obscure. Part of the mechanisms revealed in infected hepatocytes could be shared.

#### 3.1.2. HCV

Chronic HCV infection is a major risk factor for the development of HCC. HCV contains a single-stranded positive sense RNA with a single open reading frame encoding the structural proteins (core, E1, and E2), the viroporin p7, and the non-structural proteins (NS2, NS3, NS4A, NS4B, NS5A, and NS5B). Different from HBV, as an RNA virus HCV lacks a DNA intermediate phase during its life cycle. Hence, HCV infection relies on the interaction of its viral proteins with the infected hepatocytes but not the damage to the host genome [30]. Currently, the core protein NS5A and E2 have been reported to be closely related to the activation of Wnt/β-catenin signaling.

As the central component of HCV particles, the core protein is detectable in the cytoplasm, Golgi apparatus, lipid droplets, and nucleus [31,32]. Particularly, in the nucleus it potentiates the activation of Wnt/β-catenin signaling. This is achieved through increasing the expression levels of Wnt ligands, FZD, and LRP5/6 receptors [33,34], while simultaneously downregulating the transcription of Wnt antagonists SFRP2 and DKK1 [35,36]. In addition, the HCV core protein facilitates the hypermethylation at the *CDH1* gene promoter [37], leading to a reduction of E-cadherin protein expression. As a result, the β-catenin/E-cadherin complexes at the cell membrane capture less β-catenin, leading to higher levels of free β-catenin in the cytosol, thus enhancing activation of Wnt/β-catenin signaling.

As a component of the HCV RNA replication complex, NS5A enhances the ability of HCV to counteract apoptosis [38]. On the other hand, NS5A promotes Wnt/β-catenin signaling directly by binding and stabilizing the β-catenin protein [39] and indirectly by stimulating the PI3K/Akt pathway, which further mediates the inactivation of GSK3β, stabilization of β-catenin, and subsequent stimulation of β-catenin-dependent transcription [40,41,42].

HCV structural E2 protein activates the Src homology region 2 domain-containing phosphatase-2 (SHP-2) [43], which promotes Wnt/β-catenin signaling by tyrosine dephosphorylation of parafibromin. The unphosphorylated parafibromin binds and stabilizes β-catenin in the nucleus, thereby inducing target gene expression [44].

HCV enhances Wnt/β-catenin signaling independent of its proteins as well. HCV infection upregulates the expression of microRNA-155 (miR-155), which directly restrains APC expression, one of the major negative regulators in the destruction complex to regulate cytoplasmic β-catenin levels [45]. Additionally, HCV infection increases epidermal growth factor receptor (EGFR) and fibroblast growth factor (FGF) signaling, both of which lead to the release of β-catenin from the β-catenin/E-cadherin complexes as a result of tyrosine phosphorylation of β-catenin at residue Y654 and the inactivation of GSK3β through stimulation of PI3K/Akt and Ras/Raf/MEK/ERK cascades [46,47].

Apparently, HCV proteins build a network consisting of a plethora of molecular events to stimulate Wnt/β-catenin signaling, which in turn further facilitates HCV infection. Firstly, the combination of Wnt1 and Wnt5a with FZD receptors leads to the release of soluble EGFR ligands [48], which bind to EGFR triggering the co-internalization of a HCV–CD81–EGFR complex to favor HCV entry [49,50]. Secondly, Wnt/β-catenin signaling activates FGF signaling by increasing *FGF18* and *FGF20* expression [51], which enhances HCV replication and the release of infectious particles [52].

However, whether and how HCV particles regulate Wnt/β-catenin signaling in the HCV-infected cholangiocytes is still unclear.

### 3.2. Alcohol Abuse

Chronic alcohol abuse leads to alcoholic liver disease, which progresses from fatty liver through alcoholic hepatitis, hepatic fibrosis to cirrhosis, and ultimately HCC. A widely used in vivo model of chronic alcohol abuse is to feed adult male Long Evans rats with 37% ethanol for 8 weeks. In this model, nuclear and cytoplasmic expression of β-catenin was decreased in the liver, indicating that Wnt/β-catenin signaling is disrupted [53,54]. In line with this are mouse models given low ethanol concentrations within a timeframe of a few days, in which hepatic loss of β-catenin increases susceptibility to alcoholic liver disease through disrupting alcohol metabolizing enzymes, fatty acid oxidation, and fasting ketogenesis [55,56,57]. In contrast, Wnt/β-catenin signaling is activated by chronic alcohol abuse to increase hepatocyte proliferation and diethylnitrosamine (DEN)-induced tumorigenesis in a different mouse model, which requires a 4-month feeding of a 4.9% ethanol-containing diet. Ethanol increased the total number of cancerous foci and liver tumors identified in situ fixed livers from the ethanol+DEN group compared to corresponding pair-fed (PF)+DEN and chow+DEN control groups. In the ethanol+DEN group, tumor multiplicity corresponded to a 3- to 4-fold increase in proliferation and immunohistochemical staining of β-catenin in non-tumorigenic hepatocytes when compared to the PF+DEN and chow+DEN groups [58]. A similar mouse model fed a Western alcohol diet for 4 months after DEN injection was used to validate Stearoyl-CoA desaturase 2 (Scd2) as responsible for liver tumor development. Importantly, Scd2 is a target gene of Wnt/β-catenin signaling and provides a positive feedback loop to amplify the pathway via stabilization of LRP5 and LRP6 mRNA levels [59].

The paradoxical conclusions in regards to the effect of chronic alcohol abuse on the Wnt/β-catenin signaling question the optimal model for alcoholic liver disease, especially the animal strains, ethanol concentration, as well as exposure time. For humans, it takes years to develop a fatty liver without abnormal physical findings, and decades to alcoholic hepatitis (often with concomitant cirrhosis). Short-term ethanol consumption may recapitulate the early stage of alcoholic liver disease, while the 4-month mouse models may coincide with late stage. Thus, Wnt/β-catenin signaling could play a different role from the early to late stages of alcoholic liver disease, which needs further investigation.

### 3.3. Non-Alcoholic Fatty Liver Disease (NAFLD)

Non-alcoholic fatty liver disease (NAFLD), featuring fat accumulation in hepatocytes, ranges from simple steatosis to non-alcoholic steatohepatitis (NASH). The latter is associated with inflammation and fibrosis and is a major risk factor for the onset and progression of HCC [60]. From NAFLD to NASH and finally HCC, Wnt/β-catenin signaling is dynamically fine-tuned.

Aberrant adipogenesis is the central event for NAFLD, which needs the transcription factor peroxisome proliferator-activated receptor γ (PPARγ). However, Wnt/β-catenin signaling inhibits PPARγ mRNA expression [61,62]. Thus, its inactivation is required for NAFLD development, confirmed by hyperlipidemia as well as fatty liver disease resulting from non-conservative inactivating mutations in the Wnt coreceptor LRP6 in mice and by the rescue of NAFLD using Wnt ligand Wnt3A [63,64,65].

The increased burden of fat in hepatocytes, oxidative stress, and lipid peroxidation induces hepatic inflammation and fibrosis, exacerbating to NASH. During this process, Wnt/β-catenin signaling is restored by the overexpressed aortic carboxypeptidase-like protein (ACLP) that specifically binds FZD8 and LRP6 to form a ternary complex facilitating extracellular signaling transduction [66]. Levels of Wnt ligands are increased due to the complementary secretion from infiltrating macrophages [67]. Alternatively, epigenetic modifications of components involved contribute to the activation of Wnt/β-catenin signaling, including hypermethylation of Wnt antagonists, deacetylation of histones in the *AXIN2* promoter, and downregulation of microRNAs negatively regulating Wnt/β-catenin signaling [15,68].

### 3.4. Aflatoxin-B1 Exposure

Aflatoxins are a class of carcinogenic mycotoxins produced by *Aspergillus* fungi, contaminating food supplies worldwide. Aflatoxin-B1 is the most toxic aflatoxin, and it has been well validated to dramatically increase the risk of HCC in humans and animals [69]. Aflatoxin-B1 frequently induces G:C to T:A transversions at the third base in codon 249 of *TP53* and cooperates with HBV in causing p53 mutations in HCC [70]. Additionally, aflatoxin-B1 regulates Wnt/β-catenin signaling. In vitro studies using HCC cell lines treated with aflatoxin-B1 for 1 or 2 days showed that β-catenin protein levels were decreased by elevated miR-33a and miR-34a [71,72]. However, human HCCs with high exposure to aflatoxin B1 showed strong β-catenin membrane staining observed in tumor areas, compared to adjacent non-neoplastic liver tissue, possibly increasing Wnt/β-catenin signaling [73]. The discrepancy between the HCC cell lines and human HCC tissues indicates the dynamic alteration of Wnt/β-catenin signaling in the development of aflatoxin-B1-related HCC.

Accordingly, most major etiological factors of liver cancers contribute to the activation of Wnt/β-catenin signaling in precancerous lesions through multiple mechanisms, although non-virus factors suppress its activity temporarily in the early stage of pathological damage (Table 1). The elevated Wnt/β-catenin signaling enhances the proliferation of the affected liver cells, thereby overgrowing neighboring normal cells. This highlights the pivotal role of Wnt/β-catenin signaling in the transformation from precancerous lesions to liver cancers.

## 4. Liver Cancers

### 4.1. Hepatocellular Carcinoma

Around 40–70% of HCCs show β-catenin nuclear accumulation, augmenting Wnt/β-catenin signaling activity [74,75,76,77]. Mutations in key genes are involved in this process [78,79,80]. For instance, activating mutations in exon 3 of *CTNNB1* encoding β-catenin are detected in 15–25% of HCCs, producing mutated β-catenin escaping phosphorylation and subsequent degradation [8,79,81,82]. Inactivating mutations occurring in *AXIN1* are reported in 10.4% of HCCs, followed by *AXIN2* and *APC*, which are mutated in 3.3% and 1.4%, respectively [8]. The mutations in these negative regulators diminish the biological function of the destruction complex, thus favoring β-catenin accumulation. Remarkably, *CTNNB1* mutations show higher frequencies in HCV-related (26.7%) and non-viral HCCs (21.1%) than HBV-related HCCs (11.6%). Conversely, *AXIN1* mutations are more frequent in HBV-related HCCs (18%) compared to HCV-related (14%) and non-viral HCCs (8%) [82]. The difference may derive from the fact that different etiological factors cause a different local microenvironment, serving as particular selection pressure for the optimal mutation types.

However, β-catenin nuclear accumulation is recently reported to be restricted to late-stage HCC. At earlier stages, β-catenin is primarily located at the plasma membrane in complexes with multiple cadherin family members, where it drives tumor cell survival by enhancing the signaling of growth factor receptors such as EGFR [83]. This study reveals the unexpected function of β-catenin in early stages of HCC and emphasizes the complex roles β-catenin is playing during HCC progression.

In addition, other genetic and epigenetic alterations in relevant genes enhance Wnt/β-catenin signaling in liver cancer cells. Wnt ligands are elevated by excessive secretion from liver cancer cells, infiltrating macrophages as well as other cell types within cancerous foci [67,84]. In contrast, Wnt antagonists including SFRP1/4/5 and Kallistatin are decreased in HCCs, thus facilitating the combination of Wnt ligands with receptors [85,86]. On the other hand, highly expressed miR-1246 decreases expression levels of AXIN2 and GSK3β [87]. Hypermethylation in the *APC* gene leads to the loss of APC protein [88]. Together, these epigenetic alterations synergistically compromise the function of the destruction complex to promote Wnt/β-catenin signaling in HCCs.

### 4.2. Cholangiocarcinoma

Aberrant activation of Wnt/β-catenin signaling is observed in the majority of CCA, which closely associates with tumor malignancy and patient outcome. Differently from HCC, mutations in related genes are less frequent in CCA, i.e., *CTNNB1* (1.5%), *AXIN1* (4%), and *APC* (2%) [82]. Apparently, the aberrant activation seems to be more transcriptionally and epigenetically mediated. Similar to HCC, the transcription of Wnt ligands, especially Wnt7A, is increased, which is largely attributable to the secretion from macrophages present in CCA tissues [89,90]. Wnt antagonists DKK2, SFRP1, and SFRP2 are reduced as a result of promoter hypermethylation [91]. Additionally, increased expression of the retinoic acid receptor gamma and long non-coding RNA PCAT1 have been suggested to promote Wnt/β-catenin signaling in CCA [92,93]. Similar suggestions have been made for reduced expression of SOX17 and the tumor suppressor LKB1 [94,95]. 

### 4.3. Hepatoblastoma

Activation of the Wnt/β-catenin signaling pathway is deemed an important hallmark during the development of hepatoblastomas [96]. Around 60–80% of hepatoblastomas possess activating mutations in *CTNNB1*, including point mutation or deletions in exon 3 [97]. Other mutations observed occur in *APC* (20.51%), *AXIN1* (1.67%), *AXIN2* (3.75%), and *LGR6* (12.5%) [82]. Transcriptional and epigenetic modifications of related components remain unclear in the context of HB.

**Figure 2:**
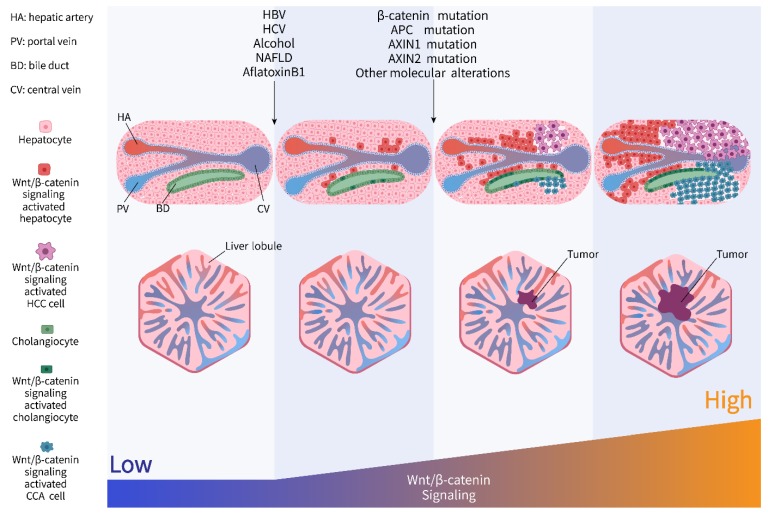
Dynamic activation of Wnt/β-catenin signaling from risk factor exposure to final liver cancer.

During the decades from risk factor exposure to final liver cancer, liver cells acquire activation of Wnt/β-catenin signaling due to the molecular interaction between etiological factors and the host. Then these cells evolve into tumor cells with robust stimulation strengthened by genomic, epigenetic, and transcriptional alterations of the components involved in Wnt/β-catenin signaling, conferring proliferative advantage and thereby contributing to the progression of tumor formation (Figure 2).

## 5. Targeting Wnt/β-Catenin Signaling in Liver Cancers

As outlined above, accumulating evidence validates the pivotal role of aberrant activation of Wnt/β-catenin signaling in the pathological process of liver cancers. It highlights potential benefits to targeting components involved in Wnt/β-catenin signaling for liver cancer treatment. During the last decades, a tremendous progression of exploring potent Wnt/β-catenin signaling inhibitors has been witnessed for various tumor types. Table 2 summarizes the reported Wnt/β-catenin signaling inhibitors tested mainly in liver cancers. These inhibitors either target the upstream molecules of Wnt/β-catenin signaling involving the PORCN protein, Wnt ligands, Wnt antagonist DKK1, FZD receptors, and LRP5/6 co-receptors, or intracellularly interfere with tankyrase activity, phosphorylation, and translocation of β-catenin as well as its interaction with co-activators or transcriptional factors. Recently, Michael et al. show that *CTNNB1* mutation in pericentral hepatocytes leads to glutamine-dependent mTORC1 activation and show a notable therapeutic benefit of mTORC1 inhibition in *CTNNB1* mutant HCC in vivo. This provides a novel way to specifically target this subset of HCCs [98].

## 6. Future Perspectives

Investigations exploring mechanisms underlying hepatocarcinogenesis accumulating over the last decades enrich our understanding of liver cancer development. Together with enormous progression in clinical technology and treatment guidelines, these achievements improve early detection and enable a more efficient treatment design for liver cancer patients. Nevertheless, liver cancer remains a major cause of tumor-related deaths worldwide.

Liver cancer patients show high phenotypic and molecular heterogeneity, contributing to the lack of a curative therapy. Correlation between phenotype and molecular characteristics is important for clinical practice in HCC patients, especially those diagnosed at an advanced stage depending on conservative treatment using small molecular inhibitors. In parallel to pathological classification, molecular profiles have been explored to categorize HCC. The group led by Jessica Zucman-Rossi proposed a distinct classification comprising six subtypes based on gene expression and have linked these subtypes to clinical and pathological classifications. Importantly, aberrant Wnt/β-catenin signaling activation is an outstanding biological feature in G5–G6, highlighting the predominant tumorigenic function of this pathway in these groups [144,145,146].

Due to its key role in HCC and other liver diseases, a large number of compounds inhibiting Wnt/β-catenin signaling have been explored. However, only a minority have been tested in liver cancer, as indicated in Table 2. These studies are mainly carried out in HCC and a few in CCA, with none in HB. In light of the critical role of Wnt/β-catenin signaling linking precancerous lesions to liver cancers, this implies the need for future investigation in non-tumor liver diseases, which is nevertheless challenging due to the dynamic regulation of Wnt/β-catenin signaling mediated by various etiological factors at different stages.

Moreover, current studies of molecules targeting components involved in Wnt/β-catenin signaling rarely consider genetic mutation or related molecular alterations. In this regard, we tested the PORCN inhibitor [128] and tankyrase inhibitor (manuscript in preparation) in HCC cell lines, some of which carry *CTNNB1* or *AXIN1* mutations. Apparently, Wnt/β-catenin signaling activity responds to these inhibitors differently depending on specific genetic defects. Despite a reduction in the signaling activity, it is not sufficient to significantly affect cell growth. These results highlight the importance of identifying the molecular features and genomic traits of liver cancer patients as well as the demand for a combined therapy to improve clinical treatment.

## 7. Conclusions

Wnt/β-catenin signaling is aberrantly stimulated during the course of liver cancer progression. The underlying mechanisms range from complicated regulation by etiological factors in precancerous lesions at early stages to acquired genomic, epigenetic and transcriptional alterations of the components involved in Wnt/β-catenin signaling in tumor foci at later stages. Therefore, targeting Wnt/β-catenin signaling potentiates a novel avenue to treat liver cancers as well as non-tumor liver diseases. However, application of molecular inhibitors targeting Wnt/β-catenin signaling for liver cancer treatment remains dim. A deeper study to explore the sensitive subgroups to these inhibitors carrying particular molecular features would aid in designing more efficient treatment strategies. Additionally, investigations targeting Wnt/β-catenin signaling in non-tumor liver diseases are still lacking, which demonstrate the need for further studies in this field.

## Figures and Tables

**Table 1:** Summary of the function of etiological factors on the regulation of Wnt/β-catenin signaling.

Etiological Factors	Roles to Regulate Wnt/β-Catenin Signaling	References
**HBV**		
HBx	♦ Downregulation of Wnt antagonist SFRP1 and SFRP5 expression due to genetic silencing by recruiting elevated DNA methyltransferase 1 and 3A to gene promoters	[20]
	♦ Disruption of the destruction complex by competitively binding APC or by inhibiting GSK3 activity through activation of Src kinase as well as induction of cell cycle-related kinase-mediated androgen receptor signaling	[21,22,23]
HBsAg	♦ Overexpression of LEF-1 and β-catenin downstream c-Myc and cyclin D1	[24,25,26]
other	♦ Insertion of HBV gene into a *LINE1* element produces an oncogenic HBV-*LINE1* chimeric transcript, inducing the nuclear localization of β-catenin.	[18,27]
**HCV**		
core protein	♦ Elevated expression levels of Wnt ligands, FZD, and LRP5/6 receptors	[33,34]
	♦ Downregulated transcription of Wnt antagonists SFRP2 and DKK1	[35,36]
	♦ Hypermethylation at the *CDH1* promoter leading to the reduction of E-cadherin and dissociation of the β-catenin/E-cadherin complexes at the cell–cell adhesion sites	[37]
NS5A	♦ Combination and stabilization of β-catenin protein	[39]
	♦ Stimulation of PI3K/Akt pathway to further inactivate GSK3β	[40,41,42]
E2	♦ Activation of SHP-2, promoting tyrosine dephosphorylation of parafibromin to bind and stabilize β-catenin in the nucleus	[43,44]
others	♦ Upregulation of miR-155 to restrain APC expression	[45]
	♦ Activation of EGFR and FGF signaling, leading to tyrosine phosphorylation of β-catenin at residue Y654 and its release from the β-catenin/E-cadherin complexes, as well as inactivation of GSK3β through PI3K/Akt and Ras/Raf/MEK/ERK cascades	[46,47]
**Alcohol abuse**	♦ Decrease of nuclear and cytoplasmic β-catenin in liver, increasing susceptibility to alcoholic liver disease in *in vivo* models treated with ethanol less than 8 weeks	[53,54,55,56,57]
	♦ Increase of β-catenin in non-tumorigenic hepatocytes in a mouse model fed a 4.9% ethanol-containing diet after DEN injection for 4 months	[58,59]
**NAFLD**	♦ Inactivation of Wnt/β-catenin signaling resulting from inactivating mutations of LRP6 in mice leads to hyperlipidemia as well as fatty liver disease, validated by rescue using Wnt3A.	[63,64,65]
	♦ Restoring of Wnt/β-catenin signaling in progression to NASH and HCC by the overexpressed ACLP, increased secretion of Wnt ligands from infiltrating macrophages, hypermethylation of Wnt antagonists, deacetylation of histones in *AXIN2* promoter and downregulation of microRNAs negatively regulating Wnt/β-catenin signaling	[15,66,67,68]
**Aflatoxin-B1**	♦ Decrease of β-catenin due to upregulation of miR-33a and miR-34a in HCC cell lines	[71,72]
	♦ Increase of β-catenin at cell membrane in HCC tissues	[73]

**Table 2:** Wnt/β-catenin signaling inhibitors undergoing preclinical and clinical evaluation in liver cancers.

Targets	Compounds	Diseases	Stage	References
FZD7	sFZD7	**HCC**	Preclinical	[99]
FZD8	OMP-54F28	**HCC**, ovarian cancer, pancreas cancer	Phase 1	[100,101,102,103,104]
LRP5/6	Salinomycin	breast, prostate, lung, gastric, osteosarcoma, **HCC**	Preclinical	[105,106,107,108,109,110]
Wnt1	Anti-Wnt1	**HCC**, CRC, lung cancer, sarcoma, breast cancer, head-neck squamous cell carcinoma	Preclinical	[111,112,113,114,115]
Wnt ligands	WIF-Fc/ SFRP-Fc	**HCC**	Preclinical	[116]
DKK1	DKN-01	**HCC**, CCA, biliary tract cancer, gallbladder cancer, and other cancers	Phase 1/2	[117,118,119,120,121,122,123,124]
PORCN	CGX1321	**HCC**, **CCA**, and other cancers	Phase 1	[125,126,127]
	IWP12	**HCC** and CRC	Preclinical	[128]
Tankyrase	XAV939/WXL-8	**HCC**	Preclinical	[129]
β-catenin phosphorylation	CGK062	CRC, **HCC**, prostate cancer	Preclinical	[130]
β-catenin	β-catenin siRNA	**HCC**	Preclinical	[131]
	BBI608	Glioblastoma, CRC, **HCC**, gastric cancer, pancreas cancer, lung cancer	Phase 1/2	[132]
β-catenin/CBP	PRI-724	Pancreatic adenocarcinoma, leukemia, CRC, **HCV-induced cirrhosis**, solid tumor	Phase 1/2	[133,134,135,136,137,138]
β-catenin/TCF	PKF115-548 PKF222-815 CGP049090 FH535	**HCC**, CRC, lung cancer	Preclinical	[139,140,141,142]
β-catenin nuclear export	Peg-IFN	**HCC**	Preclinical	[143]

The bold highlights the liver cancers, in which the compound have been tested.

## Abbreviations

HCC	Hepatocellular carcinoma
CCA	Cholangiocarcinoma
HB	Hepatoblastoma
HBV	Hepatitis B virus
HCV	Hepatitis C virus
FAP	Familial adenomatous polyposis
NAFLD	Non-alcoholic fatty liver disease
NASH	Obesity-induced non-alcoholic steatohepatitis
ER	Endoplasmic reticulum
PORCN	Wnt acyl-transferase porcupine
SFRP	Secreted frizzled-related protein
WIF	Wnt inhibitory factor
LRP	Low-density lipoprotein receptor-related protein
APC	Adenomatous polyposis coli
β-TRCP	β-transducin repeat containing protein
CRD	Cysteine rich domain
FZD	Frizzled
DVL	Disheveled
PAR	Poly-ADP-ribosylation
TNKS	Tankyrase
TCF/LEF	T-cell factor/lymphoid enhancer-binding factor
TLE	Transducin-like enhancer of split
HDAC	Histone deacetylase
HBx	Hepatitis B viral X protein
HBsAg	Hepatitis B surface antigen
LINE	Long interspersed nuclear element
SHP-2	Src homology region 2 domain-containing phosphatase-2
EGFR	Epidermal growth factor receptor
FGF	Fibroblast growth factor
DEN	Diethylnitrosamine
PPARγ	Peroxisome proliferator-activated receptor γ
ACLP	Aortic carboxypeptidase-like protein
CRC	Colorectal cancer
